# Clinical Meaningfulness of an Algorithm-Based Service for Analyzing Treatment Response in Patients with Metastatic Cancer Using FDG PET/CT

**DOI:** 10.3390/jcm13206168

**Published:** 2024-10-16

**Authors:** Manojkumar Bupathi, Benjamin Garmezy, Michael Lattanzi, Minnie Kieler, Nevein Ibrahim, Timothy G. Perk, Amy J. Weisman, Scott B. Perlman

**Affiliations:** 1 Department of Medical Oncology, Rocky Mountain Cancer Centers, Littleton, CO 80120, USA; 2 Department of Medical Oncology, Sarah Cannon Research Institute, Nashville, TN 37203, USA; 3 Genitourinary Medical Oncology, Texas Oncology, Austin, TX 78731, USA; 4 Department of Radiology, University of Wisconsin School of Medicine and Public Health, Madison, WI 53792, USA; 5 AIQ Solutions, Madison, WI 53717, USA

**Keywords:** oncology, FDG PET/CT, radiology report, clinically meaningful output, augmentative

## Abstract

**Background/Objectives**: Determining how a patient with metastatic cancer is responding to therapy can be difficult for medical oncologists, especially with text-only radiology reports. In this investigation, we assess the clinical usefulness of a new algorithm-based analysis that provides spatial location and quantification for each detected lesion region of interest (ROI) and compare it to information included in radiology reports in the United States. **Methods**: Treatment response radiology reports for FDG PET/CT scans were retrospectively gathered from 228 patients with metastatic cancers. Each radiology report was assessed for the presence of both qualitative and quantitative information. A subset of patients (*N* = 103) was further analyzed using an algorithm-based service that provides the clinician with comprehensive quantitative information, including change over time, of all detected ROI with visualization of anatomical location. For each patient, three medical oncologists from different practices independently rated the usefulness of the additional analysis overall and in four subcategories. **Results**: In the 228 radiology reports, quantitative information of size and uptake was provided for at least one lesion at one time point in 78% (size) and 95% (uptake) of patients. This information was reported for both analyzed time points (current scan and previous comparator) in 52% (size) and 66% (uptake) of patients. Only 7% of reports quantified the total number of lesions, and none of the reports quantified changes in all lesions for patients with more than a few lesions. In the assessment of the augmentative algorithm-based analysis, the majority of oncologists rated it as overall useful for 98% of patients (101/103). Within specific categories of use, the majority of oncologists voted to use it for making decisions regarding systemic therapy in 97% of patients, for targeted therapy decisions in 72% of patients, for spatial location information in 96% of patients, and for patient education purposes in 93% of patients. **Conclusions**: For patients with metastatic cancer, the algorithm-based analysis of all ROI would allow oncologists to better understand treatment response and support their work to more precisely optimize the patient’s therapy.

## 1. Introduction

For medical oncologists, deciding whether to continue the current treatment regimen for a patient with metastatic cancer or change their clinical management can be nuanced and challenging. This is because patients with metastatic cancer can have many lesions with heterogeneous response to treatment [1,2,3,4,5]. This heterogeneity adds complexity in determining whether a current treatment is effective and is compounded by factoring in individual lesions’ spatial location and rate of change. Further, oncologists must balance management of treatment-related toxicity and other symptoms with effective disease control. Oncologists have many options for changes in patient management during the response assessment period, including changes to systemic therapy, referral for radiation or other local ablative modalities, pain management, and other palliative interventions.

In current clinical practice, radiological images such as FDG PET/CT are used for diagnosing disease and assessing response to treatment. Information, both clinical and quantitative, is gathered from the images and collated in a text report by an interpreting physician. For patients with multiple lesions, the current standard practice is to measure a limited subset. Some institutions have implemented standardized response criteria such as RECIST and PERCIST [6,7]. These criteria, which are also based on quantification of a limited subset of lesions, were developed for use in clinical trials to standardize reporting, and thus were meant to show differences based on a population mean, not for individual-patient clinical decision-making [8]. Several studies have shown that extracting quantitative information from all lesions in a patient, including individual lesion rates of change, improves outcome prognosis, compared with the information from the typical subset examined in a radiology report or standardized response criteria [9,10,11]. However, this approach is currently not practical in clinical practice as it is time-consuming and subject to inter-physician variability [12]. Substantial work applying artificial intelligence to automation in oncology and PET/CT imaging has focused largely on population-level assessments and research-specific applications [13,14,15,16,17,18,19], rather than clinical implementation and providing care to individual patients.

The text-only format of standard-of-care radiology reports can be challenging for use by oncologists to completely understand a patient’s response to treatment. Written reports can result in discordance between the message intended by the radiologist/nuclear medicine physician and the message perceived by the oncologist [20]. In particular, spatial information and visualization can be difficult to convey using only written text. One study found that 86% of surveyed oncologists expressed interest in being provided with images in addition to a text-only report [21]. Also, while work is ongoing to improve the quality and consistency of radiology reports [22], oncologists report a large variation in the quality of radiology reports received in current clinical practice [23,24].

Considering both the treating physician’s need for additional information and the impracticality of that information being extracted during the typical radiology workflow, the solution to these challenges is not a technology to improve or enhance radiology reporting. Rather, the solution is to provide information that is distinct from that captured in radiology reports directly to the treating physician in a format optimized for therapeutic decision-making. This creates an opportunity for the development of a new, separate medical service that uses algorithm-based software (TRAQinform IQ v 2.0) to analyze previously acquired and interpreted serial images for interpretation and use by an oncologist. The analysis provided by this new medical service needs to present quantitative information for all detected lesions, including the rate of change across serial images. It also needs to provide information in a visual format that can be appropriately interpreted by the treating oncologist. This additional information would enable treating physicians to more effectively interpret a patient’s treatment response, discuss findings at multidisciplinary meetings, and make therapy decisions.

In this investigation, we evaluated the clinical meaningfulness of such an algorithm-based service as an addition to the standard care of patients with metastatic cancer by assessing the information needs not met by current practice as well as the clinical usefulness of an example service. To achieve this, FDG PET/CT radiology reports of patients with cancer during or after treatment were gathered and assessed for several objective criteria, aiming to capture whether the reports included basic nuclear medicine, organization, quantitative, and impression information. In a subset of patients, seven oncologists were shown results from a novel, algorithm-based, software-performed analysis and quantification capturing changes in all FDG avid regions of interest (ROI) and asked to rate its usefulness in providing patient care.

## 2. Materials and Methods

### 2.1. Data

Inclusion criteria for the study were oncology patients over the age of 22 with a previously performed pair of sequential 18F-FDG PET/CT scans as part of a standard clinical practice. Sequential scans were required to be within 18 months of one another. Queries were provided to two third-party data aggregators to ensure that the data collection included a diverse geographical and patient population.

Scans from patients with various disease indications were retrospectively collected from a variety of imaging centers in the United States. Sequential selection was performed to identify patients from varied sites with varied primary cancer types. To mitigate selection bias, the only information available during the selection process was the cancer type, scan dates (to ensure patients had pairs of sequential scans matching the eligibility criteria), and anonymized patient IDs. Radiology reports, images, and other patient information were not made available until after selection for the study.

Previously generated radiology reports of the second PET/CT scan, which were written at the time of the imaging exam by the qualified radiologist at the imaging center as part of the center’s standard-of-care protocol, were then gathered. In addition, FDG PET/CT images of the scan pairs corresponding to the two time points assessed on the radiology reports were evaluated.

### 2.2. Standard Radiology Report Analysis

A set of objective criteria for information considered important to be included in a PET scan radiology report intended for assessing treatment response was established. These criteria were grouped into four categories: nuclear medicine basics, organization, quantification, and impression. Full criteria, including explanations and clinical relevance, are shown in Table 1. The number and percentage of reports meeting each criterion are reported with 95% confidence intervals (CIs) using a test of proportions.

A separate overall “quantification” score was given to each report, defined as whether the following quantitative information was present: (1) reference region uptake *and* (2) quantification of lesion size or SUV across two time points. Reports that had this information were rated as “quantitative”. A comparison of the proportion of scans that were rated as quantitative, stratified by the median year of scan acquisition, was performed using a two-proportions test.

### 2.3. Algorithm-Based Service Analysis

Approximately half (45%) of the patients were randomly chosen for a secondary analysis. As the data was received in a randomized order, this analysis involved sequential sampling of patients with a further requirement that the sequential PET/CT scans must be collected within 12 months of one another. To minimize bias, patient information including radiology reports, disease type and stage, and imaging center were not considered in the patient selection process.

**Figure 1:**
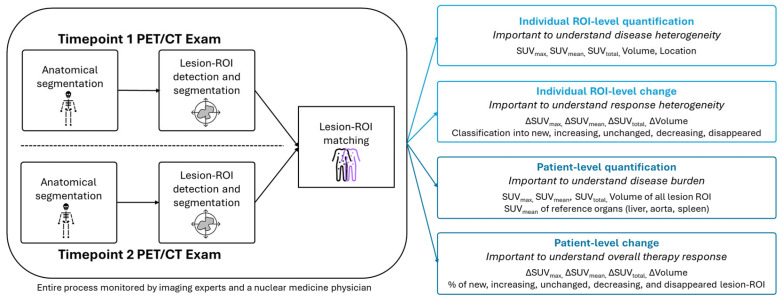
Schematic of the augmentative algorithm-based analysis including examples of output information. First, PET/CT images are segmented into 47 anatomic structures and skeleton parts using a 3D convolutional neural network methodology described in Weisman et al. [25]. Next, lesion-ROI are detected and segmented using an anatomic structure-specific PET threshold determined using a statistically optimized regional thresholding methodology outlined in Perk et al. [26]. CT images from the two time points are then deformably registered to one another before an overlap volume-based lesion matching algorithm is applied to determine which lesions are new, disappeared, or matched across scans [12]. Finally, quantitative metrics are extracted from all individual lesion-ROI and across all lesion-ROI in the patient.

Seven oncologists were asked to review the information in the radiology report, after which they were provided with an augmentative algorithm-based analysis of the serial FDG PET/CT scans to review. This analysis included a quantification of change for multiple anatomic and physiologic metrics as well as spatial location, visualization, and classification (new, increasing, unchanged, decreasing, disappeared) of each detected lesion ROI. For this study, the analysis was provided by the algorithm-based service software TRAQinform IQ (AIQ Solutions, Madison, WI, USA), which has the workflow and functionalities outlined in Figure 1. TRAQinform IQ v 2.0 is an FDA 510(k) cleared software that has been validated and established to provide excellent accuracy for anatomic structure segmentation with models previously published in Weisman et al. [25], lesion-ROI detection with methodologies similar to those established in Perk et al. [26], and a method for matching ROI across time previously assessed in multiple studies [11,12,27]. All aspects of the process are monitored by an experienced imaging expert to ensure algorithms work as expected, and by a nuclear medicine physician to ensure lesion-ROI are correctly detected and classified. The final analysis, provided in a report format that can be tailored to specific clinical needs, goes through a quality assurance review by a qualified human before it is delivered to the clinician. An example of the format used in this study is included in the Supplementary Material.

The oncologists were asked to rate the usefulness of the new, additional information provided by the software in several categories. Each patient was randomly assigned for review by three of the seven oncologists. The oncologists were asked: “Assuming you are providing care for this patient, please rate the clinical utility of the information included in the TRAQinform analysis as useful/not useful in the following categories: overall, for systemic treatment decisions, for targeted treatment decisions, for spatial location information, and for patient education”. A target of 80% of cases being rated as useful overall by the majority of oncologists was established as a threshold, indicating whether the analysis provided sufficient clinical utility. Whether this target was achieved was assessed using a two-sided test of proportions with a critical significance level of *p* = 0.05.

Separate from the oncologist assessment, the results of the algorithm-based service were collated to determine the prevalence of intra-patient response heterogeneity across the study population, similar to prior studies [5,9,10]. The presence of heterogeneity within a patient was defined as that patient presenting with at least one favorable (unchanged, decreasing, disappeared) and one unfavorable (increasing, new) ROI.

## 3. Results

### 3.1. Patient Information

A total of 228 patients from 12 imaging sites in at least 3 US states (note: exact imaging center information was anonymized prior to data collection) met the criteria for inclusion in the study. Each site had an average of 18 patients (range: 1 to 88). Demographic information for all patients is shown in Table 2. Out of the 456 total FDG PET/CT images (2 per patient), scanner information was available for 435 images (216 patients). Scans were acquired on a variety of scanner models from various manufacturers, with 186 patients receiving scans on the same scanner for baseline and follow-up. The median time between baseline and follow-up scans was 4 months (range: 1 to 18 months).

### 3.2. Standard Radiology Report Analysis

Results from the analysis of radiology report criteria are shown in Table 3. The criteria with the least frequent presence were a quantitative estimate of a number of lesions on each scan (7%) and the location of the injection site (11%). The injected dose and reason for the exam were included in all 230 reports. No reports (0/230) contained the information outlined in all 14 criteria. In all 15 reports that were noted to include quantitative information, the number of lesions noted in the report was zero (e.g., complete response to therapy). In 17 of the 151 cases (11%) where SUV was noted across the two time points, the only lesions noted in the report were new or had disappeared.

The median scan year across the gathered reports was 2018. This year was used to stratify reports into categories of early scans (before 2018) or later scans (during or after 2018). Using the binary definition of a quantitative report described previously, 3 of the 110 reports on early scans (3%) were quantitative, while 73 of the 118 reports on later scans (62%) were quantitative (*p* < 0.001).

### 3.3. Algorithm-Based Service Analysis

**Figure 2:**
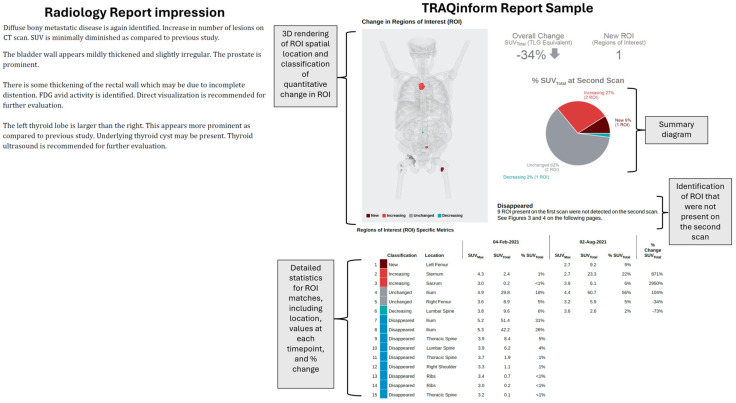
Example reports for a patient with metastatic prostate cancer (full TRAQinform report is shown in the Supplementary Materials), displaying how the augmentative algorithm-based analysis provides quantification information on all lesions that is not included in the standard radiology report.

An example of the radiology report impression section, compared with an excerpt of information from the augmentative algorithm-based service for the same patient, is shown in Figure 2. Both reports were provided to the oncologists before providing their usefulness ratings for each patient. Each oncologist provided usefulness ratings for between 36 and 50 patients, with each patient being rated by exactly three oncologists.

**Figure 3:**
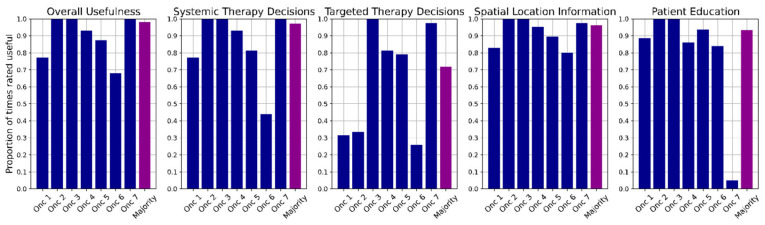
Proportion of patients reviewed by each oncologist for which the TRAQinform IQ analysis was rated as useful across the five usefulness categories. For each category, the “majority” calculation indicates the proportion of patients for which the majority of oncologists (at least two) rated the TRAQinform IQ analysis as useful.

The results of whether the oncologists viewed the information contained in the TRAQinform IQ analysis as useful across each category are shown in Figure 3. Overall, the majority of oncologists (at least two of the three) found the analysis to be useful in 101/103 patients (98.1%; 95% CI = [92.4%, 99.7%]). This was significantly above the target of 80% (*p* < 0.001). Within specific usefulness categories, the analysis was found to be useful for systemic therapy decisions in 97% (95% CI = [91.1%, 99.2%]) of patients, useful for targeted therapy decisions in 72% (95% CI = [63.0%, 80.0%]) of patients, useful for spatial location information in 96% (95% CI = [89.8%, 98.7%]) of patients, and useful for patient education in 93% (95% CI = [86.0%, 97.0%]) of patients.

Individual oncologist usefulness ratings ranged from 70–100% for overall usefulness, 38–100% for systemic therapy decisions, 23–100% for targeted therapy decisions, 83–100% for spatial location information, and 5–100% for patient education. In all categories except patient education where Oncologist 7 provided the lowest rating, Oncologist 6 provided the lowest usefulness ratings. One oncologist (Oncologist 3) provided 100% usefulness ratings for all categories. Note that direct comparisons across oncologists are difficult as each oncologist reviewed different sets of patients.

**Figure 4:**
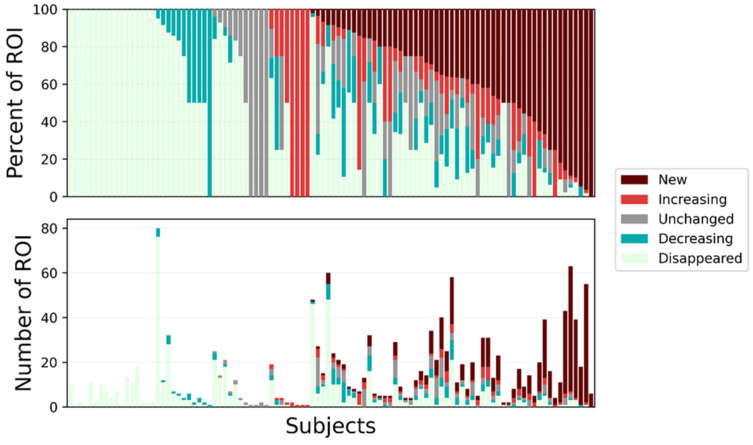
Outputs of the TRAQinform IQ analysis for all 103 patients analyzed, displaying an example of how the augmentative algorithm-based service can provide new, important information not included in the standard radiology report. In both graphs, each bar represents the distribution of lesions in each category for a single patient.

Figure 4 displays the quantification of the change of all ROI for all analyzed patients. Of the 103 patients, 56 (54%) had heterogeneity in response.

## 4. Discussion

This study suggests that important visual and quantitative information that is useful for patient care is lacking in many FDG PET/CT radiology reports, and a new algorithm-based service that offers a comprehensive quantification and analysis of change across multiple anatomic and functional parameters for each lesion ROI shows promise for improving management of patients with metastatic cancer.

The steps involved in the proposed procedure are outlined as follows. First, the oncologist determines that the patient is appropriate for the analysis and orders the service. Next, previously performed and interpreted radiological images are identified by the physician and transferred to a third party, which uses an algorithm-based service cleared by the Food and Drug Administration to perform a comprehensive quantitative analysis. This analysis is then delivered to the treating oncologist, who interprets it in combination with the previously received standard-of-care radiology reports and other patient information. As appropriate, multi-disciplinary consultation (e.g., radiology, nuclear medicine, radiation, medical, and surgical oncology) occurs, and a final treatment decision is made. This augmentative analysis would provide information that is both of clinical value to the oncologist and not available from other standard-of-care and diagnostic procedures. In this preliminary assessment, the augmentative information provided by the algorithm-based service was viewed as providing clinical utility in the vast majority of patients with metastatic solid tumors and lymphoid malignancies.

### 4.1. Clinical Usefulness of Algorithm-Based Analysis

Overall, the majority of oncologists rated the augmentative analysis as providing clinical utility in addition to the previously performed radiology report in greater than 80% of patient cases (*p* < 0.001). This may be attributed to the study’s focus on patients with metastatic disease, which resulted in a high prevalence of intra-patient response heterogeneity and patients having more than 10 lesion ROI. The rating of utility for systemic therapy decisions closely mirrored the ratings of overall clinical utility. This suggests that the additional information can help confirm both when it is appropriate to continue the current treatment and when to consider a change in systemic therapy. A change in systemic therapy could include discontinuation of the current therapy and replacement with the next line of therapy per clinical protocol, the escalation from monotherapy to combination therapy, recommendations for consideration in a clinical trial, or other approaches.

The very high rating for the utility of the spatial location information (96%) supports the hypothesis that visual information is valuable in the assessment of treatment response. A lesion located in some parts of the anatomy (e.g., the spine) may be more clinically significant than a lesion of the same size/activity in another part of the anatomy (e.g., extremities), and a visual representation of the disease can help oncologists make this assessment more efficiently and effectively. Further, oncologists reported value in being able to correlate location with the management of symptoms such as pain. Additionally, the location can influence suitability for biopsy, type of treatment (e.g., candidacy for targeted radiation), potential for grouping of lesions for targeted treatment, and prioritization of treatment.

In this investigation, “targeted therapy” was understood to refer to interventions that preferentially treat a targeted subset of lesions, such as focal therapy and surgery. The lower ratings (72%) for utility in targeted therapy decisions may be because targeted therapy might not have been appropriate in the subset of patients examined. It is likely that the greatest value to targeted therapy decisions would be in the context of oligoprogression, when a few (usually three to five) lesions show progression while the remaining metastatic disease is stable or responding. Local treatments specifically directed at resistant lesions can contribute to controlling oligoprogression, allowing the continuation of systemic treatment and potential prolongation of overall survival [28].

The very high rating of utility for patient education (93%) supports the importance of visual information as an effective tool for oncologists to use when talking with patients and their families. Patients have access to all reports in their medical records, yet the complexity of a standard radiology report can be confusing. A simple graphic to which the oncologist can refer could increase patient understanding, allowing them to more fully participate in treatment decisions and, overall, improve the patient experience. Of note, one oncologist who responded that the augmentative analysis would not be useful for patient education cited the limited time in their clinic schedule for these discussions.

### 4.2. Radiology Report Analysis

The favorable ratings for the augmentative analysis highlight the importance of adding interpretation of such an analysis to the services performed by the treating physician. In addition to the visual presentation, the most important information for oncologists includes quantification parameters (in particular, number of lesions and quantification of change in lesions) and impression of overall patient response. Only 7% of the standard radiology reports clearly quantified the number of lesions, and all of these involved patients with zero lesions at the second time point (complete response). Understanding the total number of lesions, and the change in lesions during treatment, appears to be very important for assessing response. It is also valuable for clear communication with patients. Only 52% of radiology reports quantified the change in size of a single lesion, and 66% quantified the change in SUV of a single lesion. While reports generated more recently (2018 or later) were found to be significantly more quantitative, still only 62% of those reports were quantitative, and none of the reports provided quantification of change in all lesions, as this is not standard practice. By comparison, the augmentative analysis provided by the algorithm-based service presents all of these quantified parameters, including individual assessment of each detected lesion ROI.

A surprising finding was that only 46% of the radiology reports provided a clear indication of overall patient response in the impression section. In most of the other radiology reports, the impression section simply contained a list summarizing the results, without a clear statement on overall patient response. This is consistent with previous studies showing that physicians are disappointed in the unfocused nature of radiology reports, making it difficult to determine the key message [29,30]. In addition, vague or ambiguous language has the potential to lead to poor patient care [31]. Surveys have shown that 75% of referring physicians look at the FDG PET/CT images themselves always or most of the time [32], and that only 12% of referring physicians find it uncommon to contact the interpreting physician.

Including the type of treatment is essential to provide an accurate assessment of FDG PET/CT images, especially given the possibility of treatment-specific uptake patterns such as immunotherapy-related adverse events [33], metabolic flare [34], and surgical inflammation [35]. Only 35% of radiology reports in this investigation clearly report the patient’s treatment history. However, it is possible that this information was used when interpreting the PET scan but not included in the radiology report.

While the information in the nuclear medicine basics category may be less important for oncologists clinically, it can be an indication of the overall quality of the PET scanning procedure and the radiology report by demonstrating that proper quality control and understanding of basic PET biological and technical sensitivities were considered. It was encouraging that nearly all radiology reports included patient glucose information and injected doses. On the other hand, surprisingly few radiology reports (11%) included the location of the injection site. This information is important to ensure that extravasation or other FDG activity in the arm is properly distinguished from lesions in the arm, which can be similar in size, shape, and uptake.

### 4.3. Clinical Implementation and Future Work

The range of usefulness ratings across oncologists and categories indicates that clinical adoption of such an analysis will likely vary across treating physicians and practices. It is likely that for optimal clinical meaningfulness, the results of the algorithm-based analysis would need to be organized into a report format that is personalized based on treating physician preferences and the patient population for whom the treating physician provides care. For example, one oncologist in the study (Oncologist 6) provided relatively low usefulness ratings for treatment-making decisions, but did rate the analysis as highly useful for patient education purposes. This oncologist may therefore benefit most from a more simplified version of the report specifically developed for patient education (e.g., more graphics and less text). Conversely, Oncologist 7 gave very high usefulness ratings for systemic therapy decisions while indicating they would not use the analysis for patient education. Thus, this oncologist may benefit from a report format that emphasizes metrics more relevant to changes due to systemic therapy. It is important to note that for full clinical integration of this algorithm-based analysis, all aspects of the clinical workflow must be considered including streamlining of physician requests for the analysis, image transfer, and delivery of the analysis back to the treating physician.

Although the algorithm-based analysis is independent of the standard radiology report, the information provided should be used in combination with previous radiology reports and other patient information. In their current stage of development, these analyses are not intended to diagnose disease or to replace the standard radiology report. By providing comprehensive quantitative and spatial information, the algorithm-based analysis provides new information usually not available from existing radiology reports. Also, the comprehensive lesion ROI analysis underscores the complexity of determining treatment response in the metastatic cancer patient population: 56 of 103 patients (54%) presented with at least one favorable (unchanged, decreasing, disappeared) and one unfavorable (increasing, new) ROI. However, it is important that this type of analysis be available to the oncologist to use in context with other patient data, including the radiology report, lab and biomarker analyses, patient-reported symptoms, and other information. Also, while automated technologies can identify ROI with suspected malignancy, it is important to have a qualified physician confirm the diagnosis prior to implementing an intervention. This type of analysis can also be very useful in focusing multidisciplinary discussions (e.g., tumor board).

The study was designed to determine whether the analysis provided by an augmentative, algorithm-based service has the potential to improve the management of patients with metastatic cancer. It is limited by using retrospective data for which the oncologists did not have full knowledge of the patient situations. It was also limited to the assessment of seven oncologists. The radiology report data was sourced from a variety of imaging centers in the US, and no requirements of center size or type, reporting physician experience, or other factors were provided in the data query. Thus, the data analyzed may be different from what is generated by large academic cancer centers. While the results of this study are encouraging, they should be confirmed with a prospective clinical trial. In addition, while an assessment of changes in radiology report quantification over time was performed, ideally a multivariable analysis analyzing multiple effects (e.g., report writer, imaging center, years of training) on whether a report is quantitative would be performed. Limited sample size, as well as anonymization of the exact imaging center and report writers, prevented such an analysis in this study.

## 5. Conclusions

In this investigation, a novel algorithm-based analysis provided to oncologists was shown to address quantitative and visual shortcomings of text-only radiology reports in assessing treatment response using FDG PET/CT images of patients with metastatic cancer. The majority of oncologists rated the new procedure, which provides a comprehensive quantification of change and spatial location of all ROI, as clinically useful across a variety of applications. This additional analysis would help oncologists better understand individual patients’ treatment response and support their work to more precisely optimize each patient’s therapy. The clinical meaningfulness of the algorithm-based analysis established in this retrospective study must be further confirmed in a prospective clinical trial assessing the applicability of the new analysis in various clinical settings.

## Figures and Tables

**Table 1:** Treatment response radiology report objective criteria.

Category	Information	Description and Clinical Relevance
Nuclear medicine basics	Fasting glucose levels (mg/dL)	Ensures adequate uptake of radiotracer.
	Activity of injected radiotracer (mCi)	Ensures an appropriate dose was given for adequate biodistribution.
	Quantification of reference regions (liver or blood pool)	Establishes a background value for lesion quantification.
	Location of injection site, specifically left/right arm	Aids in determining whether abnormal uptake is related to radiotracer injection or extravasation.
Organization	Separation of reported information by anatomical parts	No specific requirements of how many anatomic sections were included. Important for readability of reports.
	Reason for exam (cancer type, restaging/recurrence)	Important to ensure correct interpretation of exam.
	Patient treatment (e.g., chemotherapy, immunotherapy)	Important to ensure treatment-related effects are considered when reading the scan (e.g., immunotherapy-related adverse events).
Quantification	Numerical value for the number of lesions on the scans	Important to determine disease progression and for patient education. Note: scans marked as having “no lesions” were counted as containing this information.
	Quantification of lesion size at single time point	Numerical value required for at least one lesion, no restrictions on units (e.g., cm vs mm) required. Important to understand clinical relevance of lesion.
	Quantification of lesion size across the two time points	Two numerical values required (one for each scan) for at least one lesion unless lesion was described as new or disappeared. Important to understand change in response to therapy and clinical relevance.
	Quantification of lesion SUV at a single time point	Numerical SUV value required for at least one lesion, no restriction on type of measurement (e.g., maximum or mean). Important to understand clinical relevance of lesion.
	Quantification of lesion SUV across the two time points	Two numerical values required (one for each scan) for at least one lesion unless lesion was described as new or disappeared. Important to understand change in response to therapy and clinical relevance.
Impression	Recommended follow-up	Not important for every scan, but useful for equivocal findings.
	Clear statement on overall patient response (e.g., complete response, partial response/improvement, stable, progression, new disease)	Important to ensure scan is interpreted correctly without differences in perceived messaging.

**Table 2:** Patient and scan characteristics for all patients and scans where information was not redacted during the scan transfer process.

Cancer type, *n*	Breast cancer, *n* = 57 Lung cancer, *n* = 41 Head & neck cancer, *n* = 27 Prostate cancer, *n* = 26 Melanoma, *n* = 24	Colorectal cancer, *n* = 17 Other, *n* = 16 Lymphoma, *n* = 13 Gynecological cancer, *n* = 7
Patient sex, *n* Female/Male	113/115	
Patient age, years Median (range)	67 (25–88)	
Patient weight, kg Median (range)	76.9 (44.0–132.9)	
Patient race, *n*	Unreported, *n* = 185 White, *n* = 39 Hispanic, *n* = 2 Black, *n* = 1 Asian, *n* = 1	
Scanner model, *n*	Siemens Healthineers Biograph 20, *n* = 193 Siemens Healthineers Biograph 40, *n* = 80 Canon Medical Systems Celesteion, *n* = 69 Siemens Healthineers TruePoint (1093), *n* = 35 Canon Medical Systems Cartesion Prime, *n* = 19 GE HealthCare Discovery ST, *n* = 20 Siemens Healthineers Biograph 6, *n* = 8 Siemens Healthineers Biograph Horizon, *n* = 9 Siemens Healthineers Biograph HiRes (1080), *n* = 2 Unreported, *n* = 21	

**Table 3:** Results of presence of each criterion in the 228 radiology reports.

Category	Information	Number (Out of 228)	Percentage of Reports (%)	95% Confidence Intervals (%)
Nuclear medicine basics	Patient glucose information (mg/dL)	226	99	(96.5, 99.8)
	Injected dose (mCi)	228	100	(97.9, 100.0)
	Quantification of reference regions	97	43	(36.1, 49.3)
Organization	Location of injection site	25	11	(7.4, 15.9)
	Separated by anatomy	180	79	(73.0, 83.9)
Quantification	Reason for exam	228	100	(97.9, 100.0)
	Patient treatment	81	36	(29.4, 42.2)
	Number of lesions	15	7	(3.9, 10.8)
	Lesion size at single time point	178	78	(72.0, 83.1)
	Lesion size across two time points	119	52	(45.5, 58.8)
	Lesion SUV at a single time point	217	95	(91.3, 97.4)
	Lesion SUV across two time points	151	66	(59.6, 72.3)
Impression	Recommended follow-up	46	20	(15.3, 26.1)
	Overall patient response	105	46	(39.5, 52.8)

## Data Availability

The datasets presented in this article are not readily available because data was supplied through 3rd party data aggregators who restrict data sharing. Requests to access the datasets should be directed to tim.perk@aiq-solutions.com.

## Supplementary Materials

The following supporting information can be downloaded at: https://www.mdpi.com/article/10.3390/jcm13206168/s1, Full TRAQinform IQ Analysis.
