# Supplementary material for: Clinical Meaningfulness of an Algorithm-Based Service for Analyzing Treatment Response in Patients with Metastatic Cancer Using FDG PET/CT
Source: J Clin Med. 2024 Oct 16;13(20):6168. doi: 10.3390/jcm13206168 (PMC11508516; doi:10.3390/jcm13206168)
Supplement: Supplementary file 1 [file jcm-13-06168-s001.zip › jcm-3196333-supplementary.pdf]

Supplemental Material: TRAQinform Report Example

Informational header

Quantification of Change Analysis (FDG)

TRAQinform IQ

Name: FDG_2024CM_098 - AIQ<br>DOB: 01-Oct-1953<br>Sex: Male<br>Weight (kg): 55.3	Patient ID: 60319a868...f3a7378f31<br>Disease Type: Prostate Cancer	Scans Analyzed:<br>PET/CT - FDG 04-Feb-2021<br>PET/CT - FDG 02-Aug-2021	Report Date: 19-Jun-2024

3D rendering of ROI spatial location and classification of quantitative change in ROI

Change in Regions of Interest (ROI)

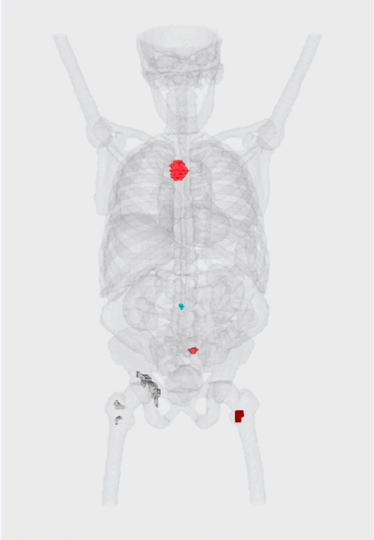

Overall Change
SUV_{Total} (TLG Equivalent)
-34%

New ROI
(Regions of Interest)
1

Summary diagram

% SUV_{Total} at Second Scan

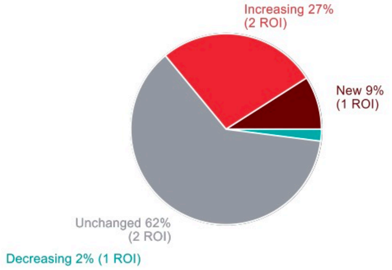

Identification of ROI that were not present on the second scan

Disappeared
9 ROI present on the first scan were not detected on the second scan.
See Figures 3 and 4 on the following pages.

Summary of ROI change categories for this scan set

Summary:
This patient underwent FDG PET/CT scans on 04-Feb-2021 and 02-Aug-2021.
Quantification and description of ROI change over time was completed using TRAQinform IQ technology.

NEW: 1 new ROI was detected in the scan on 02-Aug-2021 in the left femur representing 9% of the SUV_{Total}.

Overall, there were heterogeneous changes in the 14 ROI detected in the scan on 04-Feb-2021.

INCREASING: 2 ROI in the sternum and sacrum representing 27% of the SUV_{Total}.

UNCHANGED: 2 ROI in the ilium and right femur representing 62% of the SUV_{Total}.

DECREASING: 1 ROI in the lumbar spine representing 2% of the SUV_{Total}.

NOT DETECTED: 9 ROI in the right shoulder, ribs, thoracic spine, lumbar spine, and ilium were not detected on the scan performed on 02-Aug-2021.

PATIENT SUV_{TOTAL}: Decreased by 34%.

Report supervision information

AIQ Solutions | 8000 Excelsior Drive, Suite 400, Madison, WI 53717
+1 (608) 268-9684 ext. 109

Creation of this report was supervised by: **Nevein Ibrahim, MD**

Additional renderings of ROI spatial location and classification of quantitative change in ROI

Quantification of Change Analysis (FDG)

TRAQinform IQ

Name: FDG_2024CM_098 - AIQ<br>DOB: 01-Oct-1953<br>Sex: Male<br>Weight (kg): 55.3	Patient ID: 60319a868...f3a7378f31<br>Disease Type: Prostate Cancer	Scans Analyzed:<br>PET/CT - FDG 04-Feb-2021<br>PET/CT - FDG 02-Aug-2021	Report Date: 19-Jun-2024

Change in Regions of Interest (ROI)

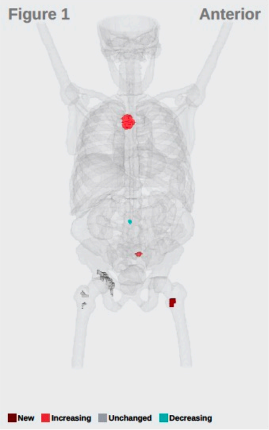

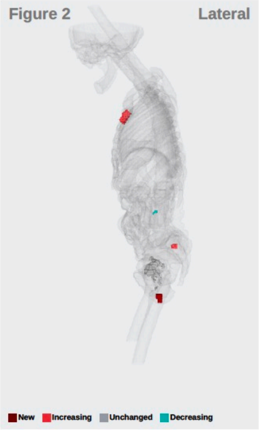

ROI Not Detected on Second Scan

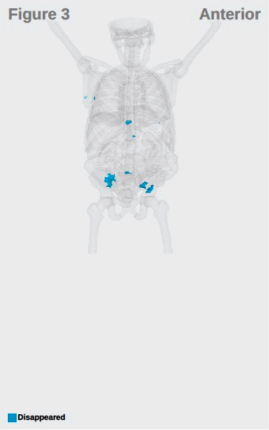

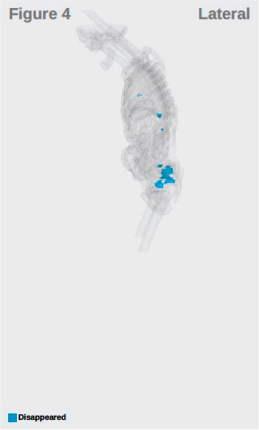

Renderings of ROI not present in second scan

Detailed statistics for reference region tracer uptake

Detailed statistics for ROI matches, including location, values at each timepoint, and % change

Quantification of Change Analysis (FDG)

TRAQinform IQ

Name: FDG_2024CM_098 - AIQ
DOB: 01-Oct-1953
Sex: Male
Weight (kg): 55.3

Patient ID: 60319a868...f3a7378f31
Disease Type: Prostate Cancer

Scans Analyzed:
PET/CT - FDG 04-Feb-2021
PET/CT - FDG 02-Aug-2021

Report Date: 19-Jun-2024

Reference Region Tracer Uptake Table

Region	04-Feb-2021 SUV_{Mean}	02-Aug-2021 SUV_{Mean}
Liver	2.1	1.4
Aorta	1.7	1.2
Spleen	1.7	1.4

Regions of Interest (ROI) Specific Metrics

	Classification	Location	04-Feb-2021 SUV_{Max}	04-Feb-2021 SUV_{Total}	04-Feb-2021 % SUV_{Total}	02-Aug-2021 SUV_{Max}	02-Aug-2021 SUV_{Total}	02-Aug-2021 % SUV_{Total}	% Change SUV_{Total}
1	New	Left Femur				2.7	9.2	9%	
2	Increasing	Sternum	4.3	2.4	1%	2.7	23.3	22%	871%
3	Increasing	Sacrum	3.0	0.2	<1%	3.9	6.1	6%	2950%
4	Unchanged	Ilium	4.9	29.8	18%	4.4	60.7	56%	104%
5	Unchanged	Right Femur	3.6	8.9	5%	3.2	5.9	5%	-34%
6	Decreasing	Lumbar Spine	3.8	9.6	6%	3.6	2.6	2%	-73%
7	Disappeared	Ilium	5.2	51.4	31%				
8	Disappeared	Ilium	5.3	42.2	26%				

AIQ Solutions | 8000 Excelsior Drive, Suite 400, Madison, WI 53717
+1 (608) 268-9684 ext. 109

Creation of this report was supervised by: **Nevein Ibrahim, MD**

Detailed statistics for ROI matches, including location, values at each timepoint, and % change

TRAQinform IQ

Name: FDG_2024CM_098 - AIQ<br>DOB: 01-Oct-1953<br>Sex: Male<br>Weight (kg): 55.3	Patient ID: 60319a868...f3a7378f31<br>Disease Type: Prostate Cancer	Scans Analyzed:<br>PET/CT - FDG 04-Feb-2021<br>PET/CT - FDG 02-Aug-2021	Report Date: 19-Jun-2024

Regions of Interest (ROI) Specific Metrics Continued

	Classification	Location	04-Feb-2021			02-Aug-2021			% Change
			SUV_{Max}	SUV_{Total}	% SUV_{Total}	SUV_{Max}	SUV_{Total}	% SUV_{Total}	SUV_{Total}
9	Disappeared	Thoracic Spine	3.9	8.4	5%				
10	Disappeared	Lumbar Spine	3.9	6.2	4%				
11	Disappeared	Thoracic Spine	3.7	1.9	1%				
12	Disappeared	Right Shoulder	3.3	1.1	1%				
13	Disappeared	Ribs	3.4	0.7	<1%				
14	Disappeared	Ribs	3.0	0.2	<1%				
15	Disappeared	Thoracic Spine	3.2	0.1	<1%				

Metrics, acronyms and definitions

Quantification of Change Analysis (FDG)

TRAQinform IQ

Name: xxxx xxxx	Patient ID: xxxx	Scans Analyzed:	Report Date: 12-Apr-2024
DOB: 11-Jan-1960	Disease Type: Lung Cancer	PET/CT - FDG 14-Feb-2014	
Sex: M		PET/CT - FDG 08-Jun-2014	
Weight (kg): 80			

Metrics, Acronyms, and Definitions

METRIC	DEFINITION	CLINICAL INDICATION
ROI	Region of Interest	Volume of potential interest based on imaging analysis
SUV	Standardized Uptake Value	Quantification of measured radioactivity
SUV_{Total} (TLG Equivalent)	SUV_{mean} × ROI volume calculated at both ROI and patient level. Values are presented in units of g/mL × cm^3.	Global indication of ROI metabolism
SUV_{Max}	Highest SUV within an ROI	Measure of the highest glucose metabolism in the ROI
TLG	Total Lesion Glycolysis	Global indication of ROI metabolism
Unclassifiable	Unclassifiable ROI were those not completely visualized on both imaging scans preventing an accurate comparison and classification	Quantitative data that is Unclassifiable may represent only a portion of the ROI. A more comprehensive scan may be necessary to fully assess that ROI.

Technology description

Quantification of Change Analysis (FDG)

TRAQinform IQ

Name: FDG_2024CM_098 - AIQ	Patient ID:	60319a868...f3a7378f31	Scans Analyzed:		Report Date:	19-Jun-2024
DOB: 01-Oct-1953	Disease Type:	Prostate Cancer	PET/CT - FDG	04-Feb-2021		
Sex: Male			PET/CT - FDG	02-Aug-2021		
Weight (kg): 55.3						

TRAQinform IQ provides quantitative analysis of CT and PET/CT images and is intended for use by trained medical professionals as a clinical decision support tool. TRAQinform IQ is not intended to diagnose any disease, replace the diagnostic procedures for interpretation of CT or PET/CT images, recommend any specific treatment, nor replace the skill and judgment of qualified medical professionals.

Certain biological and/or clinical factors including treatment history and imaging protocols can impact the interpretation of this analysis. The regions of interest identified by the technology are indicative only and may or may not equate to cancerous lesions. No imaging based, non-invasive technology can determine categorically that regions of interest identified are cancerous lesions. AIQ's technology uses a proprietary combination of trained AI models, voxel thresholding and heuristics to identify ROIs. Contact info@aiq-solutions.com for more information on the validation of AIQ's technology.

Decisions on patient care and treatment must be based on the independent medical judgment of the treating physician, taking into consideration all applicable information concerning the patient's condition, such as patient and family history, physical examinations, information from other diagnostic tests, and patient preferences, in accordance with the standard of care in a given community.

THE TRAQINFORM IQ REPORT IS PROVIDED ON AN "AS-IS" BASIS. AIQ HEREBY DISCLAIMS ANY AND ALL WARRANTIES, WHETHER EXPRESS, IMPLIED, STATUTORY OR OTHERWISE. AIQ SPECIFICALLY DISCLAIMS ANY WARRANTY OF MERCHANTABILITY OR WARRANTY OF FITNESS FOR A PARTICULAR PURPOSE, WHETHER EXPRESS OR IMPLIED BY LAW, COURSE OF DEALING, COURSE OF PERFORMANCE, USAGE OF TRADE OR OTHERWISE. AIQ SHALL NOT BE LIABLE FOR ANY DIRECT, INCIDENTIAL, OR CONSEQUENTIAL DAMAGES.

Feedback
Scan Me
